# High-Resolution CT Findings of Myositis-Related Interstitial Lung Disease

**DOI:** 10.3390/medicina57070692

**Published:** 2021-07-06

**Authors:** Ryoko Egashira

**Affiliations:** Department of Radiology, Faculty of Medicine, Saga University, 5-1-1 Nabeshima, Saga 849-8501, Japan; egashira@cc.saga-u.ac.jp; Tel.: +81-952-34-2309; Fax: +81-952-34-2016

**Keywords:** interstitial pneumonia, interstitial lung disease, polymyositis/dermatomyositis, anti- synthetase syndrome, high-resolution CT, myositis-specific antibody

## Abstract

Myositis-related interstitial lung disease presents with a wide variety of lesions, ranging from chronic to acute. It can be divided into two main forms by the types of onsets, namely, chronic to subacute type showing nonspecific interstitial pneumonia (NSIP) or NSIP with an organizing pneumonia (OP)/fibrosing OP (FOP) pattern and acute type showing acute lung injury (ALI) to diffuse alveolar damage (DAD) pattern. Anti-aminoacyl tRNA Synthetase antibody-positive cases mainly show an NSIP or FOP pattern, whereas anti-melanoma differentiation-associated gene 5 antibody-positive cases show ALI to DAD pattern. Bilateral consolidation with or without ground-glass opacification with lower lobe predominance is common as a major pattern in all types, but the distribution or extent is sometimes different. The early detection of findings that indicate a rapid progressive course is vital. Diffuse cranio-caudal distribution and multiple ground-glass opacifications with random distribution might indicate a poorer prognosis.

## 1. Introduction

Pulmonary manifestations associated with polymyositis/dermatomyositis (PM/DM) includes interstitial lung disease (ILD), ventilation defects caused by an involvement of respiratory muscle, and aspiration pneumonia resulting from pharyngeal muscle involvement. Only ILD will be discussed here, but the other manifestations could co-exist with ILD in clinical practice and should be considered as possible components of parenchymal lesions.

Myositis-related ILD includes PM/DM-related ILD and ILD associated with myositis-specific antibodies. Previously, it was common to examine high-resolution computed tomography (HRCT) findings on a disease basis, but with the discovery of a number of autoantibodies, it has been reported that different antibodies correspond to different imaging findings. Patients with positive myositis-specific autoantibodies often have the same CT findings as patients with the same antibodies, even if they do not have myositis, and may later develop myositis.

In this review, (1) HRCT features of myositis spectrum disease-related ILD (including myositis-specific antibody-positive ILD without myositis such as anti-synthetase syndrome (ASS)) and (2) HRCT findings related to poor prognosis that should be considered in treatment, will be discussed including the latest findings.

## 2. HRCT Features of Myositis Spectrum Disease-Related ILD Based on Clinical Course

**Figure 1:**
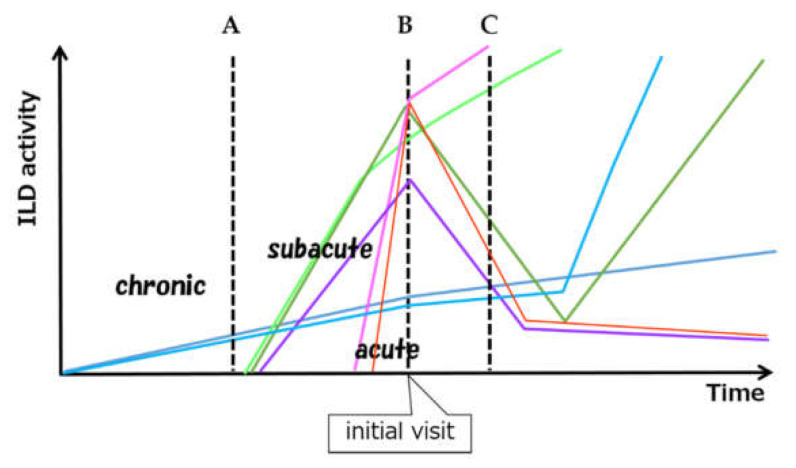
Schematic view of considerable variation of ILD activity and clinical course. A, B and C mean the time of the diagnosis of myositis. A: Subclinical onset of chronic type of ILD found at the time of the diagnosis of myositis. B: Simultaneous onset and diagnosis of acute/subacute type of ILD C: Preceding ILD and delayed onset of myositis. ILD can develop subclinically, can relapse after remission. (ILD: interstitial lung disease).

Myositis-related ILDs commonly present as chronic active inflammatory lesions or as acute lung injury. Many combinations of ILD are possible based on onset type, activity, and timing of myositis diagnosis, as well as a wide variety of subsequent outcomes (Figure 1).

**Figure 2:**
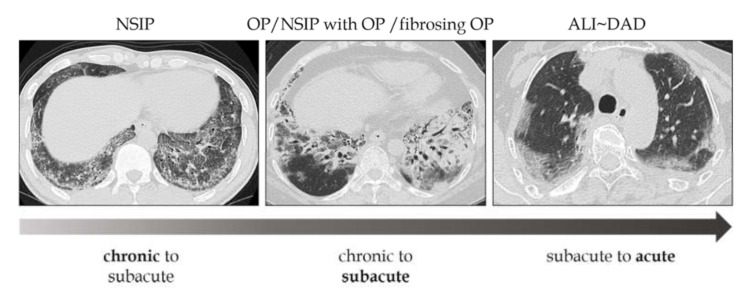
Relationship between morphologic patterns and the types of onset. The phenotypes of these lesions are difficult to distinguish clearly, given that varying degrees of inflammation and lung injury exist on the same spectrum. NSIP: nonspecific interstitial pneumonia, OP: organizing pneumonia, ALI: acute lung injury, DAD: diffuse alveolar damage.

The phenotypes of these lesions are difficult to distinguish clearly, given that varying degrees of inflammation and lung injury exist on the same spectrum (Figure 2).

**Figure 3:**
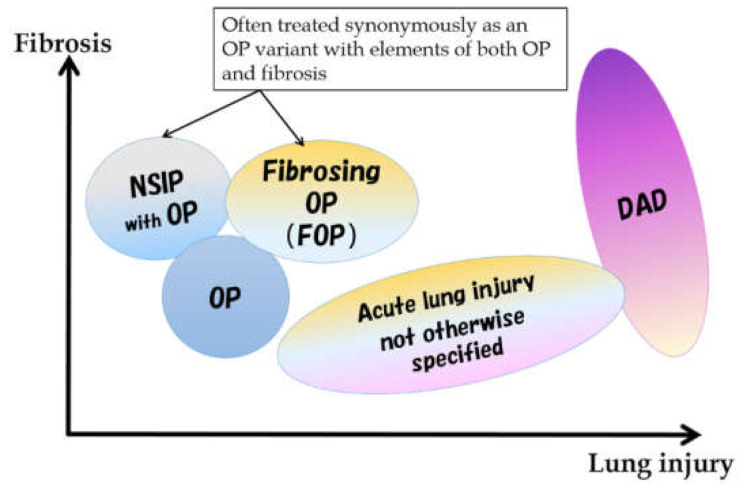
Spectrum of morphological patterns in subacute to acute pathologies. NSIP: nonspecific interstitial pneumonia, OP: organizing pneumonia, DAD: diffuse alveolar damage.

Although the majority of PM/DM-related ILDs fall on the spectrum of subacute to acute lesions at the onset, some may be difficult to explain using existing classifications alone. Histologically, the subacute to chronic form shows a nonspecific interstitial pneumonia (NSIP) pattern or an organizing pneumonia (OP) pattern [1,2], but a mixed NSIP and OP pattern is also common. Among the findings previously reported as NSIP patterns, a pattern called fibrosing OP (FOP) or OP with fibrosis in the current classification [3] is also included. The FOP pattern has been proposed as an intermediate pathology between NSIP and OP [3,4]. It corresponds to OP with a stronger fibrosing nature that is difficult to eliminate with steroids alone and leads to fibrotic ILD. Conversely, the acute form has an acute lung injury (ALI) pattern ranging from a diffuse alveolar damage (DAD) pattern to a slightly milder ALI pattern (Figure 3). A usual interstitial pneumonia pattern was also reported, but it would be an end-stage pathology of the above.

The rapidly progressive, acute type is more common in DM than in PM [5]. The general prognosis is poor, especially in patients with anti-melanoma differentiation-associated gene 5 (anti-MDA5) antibody, as nearly half of them have rapidly progressive interstitial pneumonia [6,7]. The relationship between antibodies and imaging findings will be discussed further on in the article.

Another characteristic feature of PM/DM is the high frequency of ILD that precedes muscle and skin lesions [8]. Some cases require time to make a definite clinical diagnosis, whereas some cases develop ILD alone as a preceding lesion [9]. In cases of PM/DM-ILD with rapid progression, the initial treatment is important. Furthermore, the decision on the first treatment affects the prognosis. Therefore, considering the possibility of myositis-related ILD with rapidly progressive course based on HRCT findings is desirable.

In myositis-related ILD, ground-glass opacification (GGO), consolidation, traction bronchiectasis, irregular linear opacities and bronchovascular thickening are common and frequent, and no difference can be identified in the findings among the myositis subtype (PM, DM, or clinically amyopathic DM) [10]. However, differences can be observed in the imaging findings between the chronic/subacute and acute forms of the disease. The details are described below, and the summary is shown in Table 1.

### 2.1. Subacute to Chronic Type

**Figure 4:**
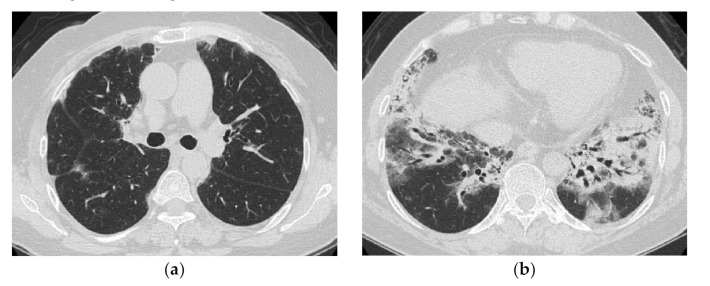
A 50-year-old woman with subacute onset interstitial lung disease associated with dermatomyositis. There is a bilateral basal consolidation extending along the bronchovascular bundles with mild ground-glass opacification and mild traction bronchiectasis within (**b**). Patchy subpleural consolidation is also seen at the level of the pulmonary artery trunk (**a**).

Pulmonary lesions with a subacute to chronic course are characterized by a combination of GGO, consolidation and reticular opacities, with concomitant findings of fibrosis such as traction bronchiectasis and volume loss [11,12,13]. The distribution can be roughly divided into the following: (1) bilateral consolidation with or without GGO with predominance of peribronchovascular bundles in lower lobes (mainly, seen in subacute cases); (2) multiple patchy consolidation in the subpleural area (seen in subacute to chronic cases); (3) reticulation and GGO spreading with subpleural and lower predominance (mainly, seen in chronic cases). Consolidation with GGO, traction bronchiectasis, and volume loss with peribronchovascular and lower lung predominance are the most frequent and typical findings [11,13] (Figure 4).

**Figure 5:**
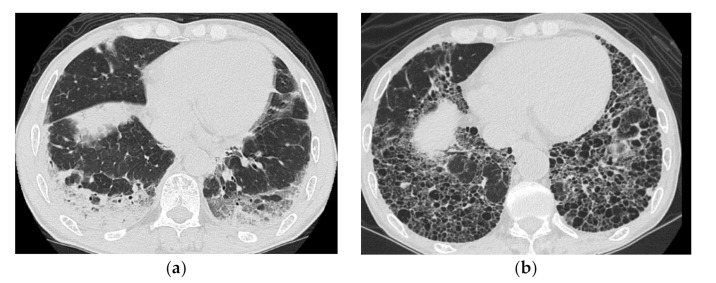
A 51-year-old man with subacute onset interstitial lung disease associated with polymyositis. At the time of initial diagnosis (**a**), there was a subpleural dense consolidation to ground-glass opacification, accompanied by a decreased lower lobe volume. Then, 13 years later (**b**), the lesion became more extensive, and the areas of consolidation had changed as an area with reticular and small cystic lesion, some of which resembled a honeycomb lung.

Histologically, these lesions correspond to diffuse thickening of the alveolar septa with fibrosis, and inflammatory cell infiltration (NSIP pattern), and are often accompanied by immature polypoid organizations protruding into the lumen (OP pattern). Akira et al. [14] reported that a patient with PM/DM who initially showed subpleural consolidation and histological bronchiolitis obliterans organizing pneumonia (included in the current OP) on CT showed honeycomb-like findings on follow-up CT (Figure 5). Cases like this would be classified as FOP.

The response to treatment is initially relatively good, and the GGO and consolidation tend to disappear. However, fibrotic lesions such as traction bronchiectasis and volume loss usually persist. Relapses during the course of the disease and sudden acute exacerbations can also occur.

**Figure 6:**
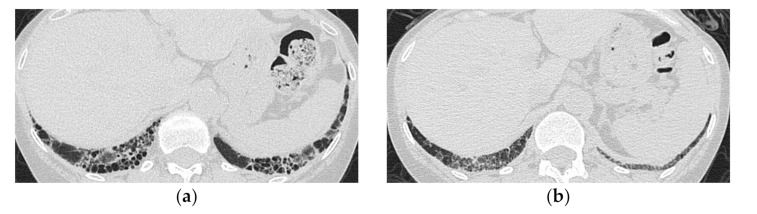
A 59-year-old woman with dermatomyositis under long-term follow-up. The most recent high-resolution computed tomography (HRCT) (**a**) shows bilateral honeycomb-like cystic lesion just below the pleura, and a review of the HRCT scan at the time of initial presentation 17 years ago (**b**) shows that the same area was previously ground-glass opacification with mild reticulation showing NSIP pattern.

In the literature, UIP pattern showing honeycomb formation with basal predominance have also been reported [11,12,15]. In fact, in cases with a long-term follow-up, a cluster of small cystic lesions in the subpleural area of the basal lung has often been observed, presenting like a honeycomb. However, a retrospective review of previous CT images suggests that the honeycomb-like appearance is more likely to be a GGO or consolidation in nature (Figure 6). UIP pattern in myositis-related ILD would be an end-stage pathology of NSIP and/or OP pattern. Additionally, Aggarwal et al. reported that myositis-associated UIP pattern cases showed a better prognosis than IPF [15]. The pathogenesis of the lesion might differ from that of the UIP pattern in idiopathic pulmonary fibrosis.

### 2.2. Acute Type

**Figure 7:**
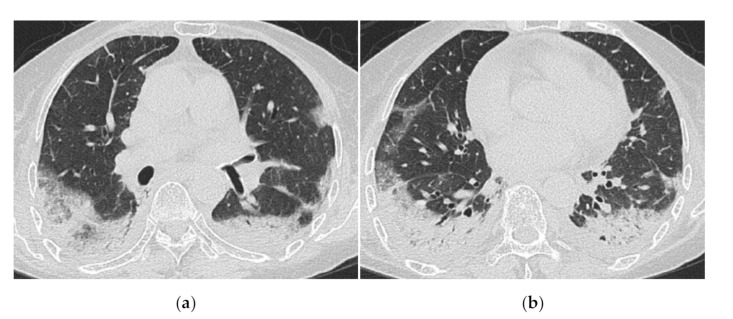
A 70-year-old woman with rapidly progressive interstitial lung disease associated with clinically amyopathic dermatomyositis (antibody unknown). HRCT images (**a**,**b**) show diffuse ground-glass opacification and consolidation just below the pleura. Crazy-paving appearance, interlobular septal thickening and bilateral pleural effusions are also seen. The patient died 2 weeks after admission.

The acute form of the disease is more common in DM than in PM. They often present as rapidly progressive ILD at onset, and the prognosis is often poor. Diffuse multiple GGO and consolidation are seen, and consolidation is often distributed around the bronchovascular bundle of the lower lobes [16,17], corresponding to the DAD pattern. A faint increasing in attenuation may be observed in apparently normal areas, which reflect diffuse edematous changes, or GGO and overlapping interlobular/intralobular interstitial thickening (crazy-paving appearance), which reflect permeability edema in the pulmonary parenchyma (Figure 7).

## 3. Association between Myositis-Specific Antibodies and ILD

Myositis-specific antibodies includes anti-aminoacyl tRNA Synthetase antibody (Anti-ARS Ab), anti-MDA5, anti-SRP, anti-TIF-1γ and other several antibodies. Here, we only discuss the HRCT findings associated with anti-ARS and anti-MDA5 antibodies.

### 3.1. Anti-Aminoacyl tRNA Synthetase Antibody (Anti-ARS Ab)

Eight types of anti-aminoacyl tRNA synthetase antibody (anti-ARS Ab) have been identified, including the well-known anti-Jo-1 antibody and anti-PL7, anti-EJ, anti-OJ, anti-PL12, and anti-KS antibodies. Anti-ARS antibodies are found in both PM and DM and are closely related to one another. However, antibody positivity is not synonymous with myositis and may also be seen in other collagen diseases and idiopathic interstitial pneumonia [18].

Anti-ARS Ab is a general term for several antibodies, and although the clinical picture differs slightly depending on the antibody, the frequency of ILD is generally high (more than 90%). Other clinical symptoms such as polyarthritis, Raynaud’s phenomenon, and mechanical hand are also frequently observed. It is referred to as anti-synthetase syndromes (ASS) independent of myositis.

According to a study by Pinal-Fernandez et al. [19] of 169 patients with ASS, the frequency of ILD at disease onset and the severity of lung lesions were higher in patients with anti-PL7 and anti-PL12 antibodies. Patients positive for anti-Jo-1 antibodies had a slightly lower frequency of ILD, but a higher frequency of muscle symptoms. In patients with anti-PL7 and anti-PL12 antibodies, the frequency of pulmonary lesions but no muscle symptoms (clinically amyopathic DM (CADM) or idiopathic interstitial pneumonia (IIPs) without myositis) was as high as 19% and 30%, respectively. Pulmonary involvement in ASS has a subacute to chronic course and is often more responsive to initial therapy than in MDA5 Ab-positive cases. Nonetheless, relapses during the course of the disease are common [20].

#### HRCT Findings of Patients with Anti-ARS Ab

**Figure 8:**
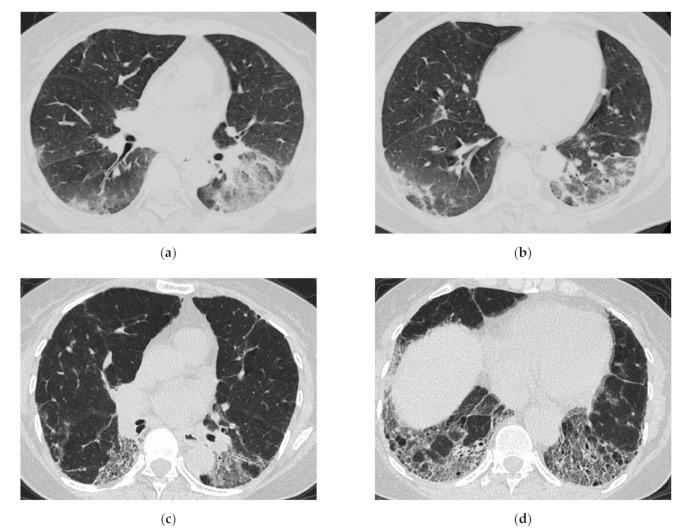
A 64-year-old woman with subacute onset interstitial lung disease associated with polymyositis. At the time of initial diagnosis (**a**,**b**), there was bilateral consolidation to ground-glass opacification with lower and peribronchovascular predominance. Then, 13 years later (**c**,**d**), the lesion became less extensive, and the lesion had changed into reticulation with traction bronchiectasis. Decreased lung volume of the lower lobes was visible.

Waseda et al. [18] evaluated the CT findings of 64 patients with ILD (including PM/DM, other connective tissue diseases, and IIPs) presenting with ASS. The most common lesions were GGO (98.4%), followed by reticular opacities (67.2%) and consolidation (48.4%). Notably, the distribution of these findings is relatively homogeneous regardless of the background disease, considering they are mainly found in the lower lung fields (98.4%), around the bronchovascular bundles (73.4%), and the periphery (95.3%), accompanied by a loss of volume in the lower lobes (89.1%). In terms of pattern classification, the NSIP pattern accounted for the largest number of cases (*n* = 35), followed by the FOP pattern (*n* = 22), which is easier to understand if we refer to the FOP pattern as the consolidation pattern of the former NSIP pattern (Figure 8).

Hozumi et al. [21] investigated positivity for myositis-specific antibodies in PM/DM-ILD and found that all CT findings in patients positive for anti-ARS Ab indicated an NSIP pattern or NSIP with OP pattern.

**Figure 9:**
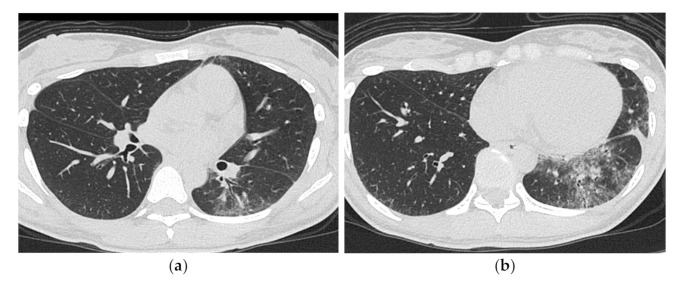
A 30-year-old man with nonspecific interstitial pneumonia pattern associated with dermatomyositis (anti-ARS Ab positive). HRCT images (**a**,**b**) shows bilateral ground-glass opacification and reticulation with peribronchovascular predominance. Mild traction bronchiectasis and decreased volume in the left lower lobe was also seen.

Some studies have investigated anti-Jo-1 antibodies-related ILD, which have been available for general clinical use since early times. Karadimitrakis et al. [22] found that the CT findings of 17 patients with anti-Jo-1 antibody-positive PM/DM, 14 of whom had ILD, showed a predominance of GGO and reticular opacities in the lower lung fields, sometimes with traction bronchiectasis consistent with the NSIP pattern. Tillie-Leblond et al. [17] studied 32 patients with anti-Jo-1 antibody-positive ASS with ILD, divided into acute/subacute-onset and slow-onset groups, and found that diffuse and patchy GGO and the combination of diffuse GGO, irregular linear shadows with predominance in the lung base, and consolidation with predominance in the lung base were significantly more common in the acute/subacute-onset group than in the slow-onset group (Figure 9).

### 3.2. Anti-MDA5 Antibody (Anti-MDA5 Ab)

The anti-MDA5 antibody was initially called the anti-CADM-140 antibody, since it was first found in patients with CADM who were clinically asymptomatic for myositis. It was renamed after MDA5 was found to be the corresponding antigen [7].

Patients who are positive for Anti-MDA5 Ab usually present with a rapidly progressive acute to subacute course of the disease, and often present to the emergency department with severe dyspnea. The prognosis of Anti-MDA5 Ab-positive patients is determined by the ability to save the patient in the early stages of the disease [21]. When the patient presents with rapidly progressive ILD, a diagnosis for DM may not be possible to make clinically. Notably, the indication of Anti-MDA5 Ab positivity based on imaging findings may be lifesaving.

#### HRCT Findings of Patients with Anti-MDA5 Ab

Since Anti-MDA5 Ab had only been discovered a short time ago, reports on CT findings are few. Basically, Anti-MDA5 Ab positivity is thought to present as an ALI with a rather severe OP pattern leading to DAD but without chronic lesions.

**Figure 10:**
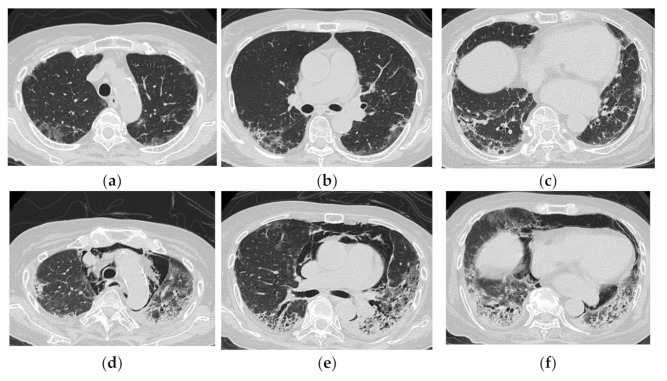
An 83-year-old woman with dermatomyositis related interstitial lung disease (anti-MDA5 Ab positive). At the time of initial diagnosis (**a**–**c**), there was diffuse ground-glass opacification and consolidation with basal and subpleural predominance. Subpleural parenchymal band-like opacity was also seen. One month later (**d**–**f**), the lesion became more extensive, and increase of reticulation with traction bronchiectasis, lower volume loss was visible. Note the severe mediastinal emphysema.

Tanizawa et al. [23] evaluated the CT images of 51 patients with PM/DM-ILD at the time of initial diagnosis in three groups and found that those with a lower consolidation/GGO (consolidation or GGO in the peripheral lower lung fields or along the bronchovascular bundles) were more likely to be anti-CADM-140 Ab-positive. Tanizawa et al. also compared the HRCT findings of 25 patients with DM-associated ILD, who were divided into anti-CADM-140 Ab-positive (*n* = 12) and anti-CADM-140 Ab-negative (*n* = 13) groups [16]. The HRCT patterns differed significantly between the two groups. In patients positive for anti-CADM-140 Ab, GGA was more frequently seen, and cranio-caudal distribution was more extensive than in the anti-CADM-140 Ab-negative group. In addition, they suggested that HRCT evaluation can help predict anti-CADM-140 Ab positivity. Four patients with anti-CADM-140 Ab-positive CADM were all found to have lower lobe-predominant consolidation and GGO, and one of them had DAD confirmed by pathological examination [24] (Figure 10).

**Figure 11:**
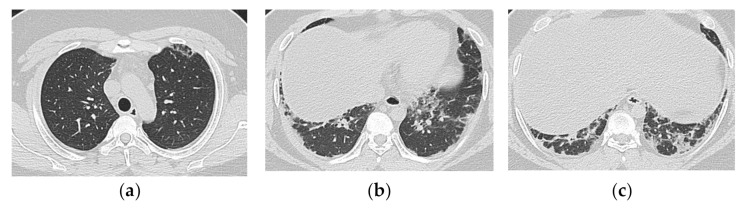
A 19-year-old man with interstitial lung disease associated with dermatomyositis (anti-MDA5 Ab positive). On HRCT (**a**–**c**), bilateral subpleural patchy consolidation are seen with basal predominance. Peribronchovascular component is not predominant. The onset was subacute, but the clinical course was stable.

Anti-MDA5 positivity is recognized as a poor prognostic factor and relatively aggressive treatment is sometimes chosen. This may or may not be the reason, anti-MDA5 antibody positive cases with a relatively good prognosis are also becoming more common (Figure 11). The key findings should be explored in order to pick up truly rapidly progressive cases.

The summary of findings based on the types of antibodies is shown in Table 2.

## 4. HRCT Findings in Relation to the Poorer Prognosis

Bonnefoy et al. [12] assessed the serial changes in patterns seen on HRCT. They classified the initial HRCT pattern into four groups: GGO and reticulation (45%), consolidation (20%), honeycomb (20%), and normal or almost normal (15%). They concluded that clinical deterioration and DAD would be observed during the follow-up period, regardless of the HRCT pattern demonstrated at the initial visit, and that predicting the course of the disease from the initial CT findings is difficult.

**Figure 12:**
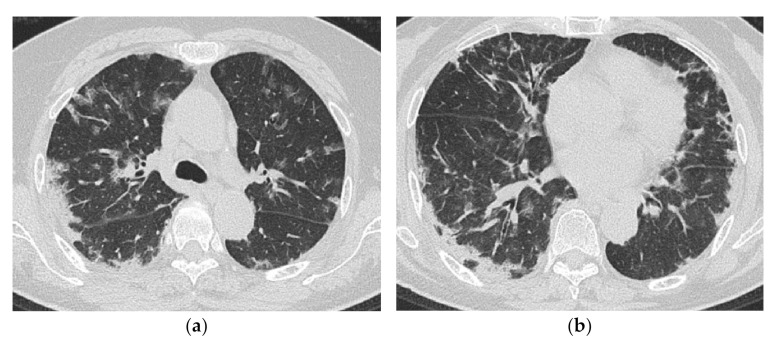
A 69-year-old woman with rapidly progressive interstitial lung disease associated with dermatomyositis (antibody unknown). On HRCT (**a**,**b**), bilateral diffuse patchy ground-glass opacification and consolidation are seen in both lungs. The distribution of the lesions is unbiased both in the vertical and horizontal directions. A transbronchial lung biopsy specimen showed diffuse edematous thickening of the alveolar septa and fibrin deposition, which was considered to correspond to acute lung injury (not shown). The patient died three weeks after admission.

However, similarities were observed in the CT findings of poor prognosis cases reported in several papers. Akira et al. [14] analyzed seven patients, including two fatal cases with PM/DM-ILD, and reported that one fatal case showed multifocal patchy consolidation and GGO, which was proven to be DAD at autopsy. Mino et al. [25] reported CT findings of 19 patients with PM/DM-ILD, together with their serial changes. In this case series, one of the patients who died due to progression of lung lesions presented with a randomly distributed GGO. Tillie-Leblond et al. [17] also observed a high incidence of diffuse patchy GGO in a group of anti-Jo1 Ab-positive myositis patients with a subacute/acute onset, resulting in two deaths out of 15 patients. Randomly distributed GGO could be a sign of poor prognosis, which thus requires a stronger treatment (Figure 12).

Zou et al. examined the CT findings of 37 CADM patients at initial presentation using the HRCT score developed by Ichikado et al. [26]. An HRCT score of 182 or higher was identified as a risk factor of 1-year mortality [27].

## 5. Clinical Pearls from HRCT Findings

In cases with a diagnosis of myositis, the role of HRCT is to determine the extent of the disease, the degree of fibrosis and to capture rapidly progressive disease. More aggressive treatment and careful follow-up are desirable when HRCT shows extensive lesion, multiple GGOs with random distribution, and a typical DAD pattern. Even in cases where myositis is not clinically evident, it is advisable to proceed with antibody testing for the possibility of myositis-related lesions when encountering patients with an OP to DAD pattern on HRCT.

Many types of viral pneumonia share HRCT features with subacute to acute type myositis-related ILD [28,29,30]. In view of the ongoing COVID-19 pandemic caused by SARS-CoV-2 infection, the possibility of viral pneumonia or complications of infection should always be considered in the presence of such lesions. At the same time, the fear of SARS-CoV-2 virus infection should not delay the diagnosis of rapidly progressive ILD requiring early therapeutic intervention.

## 6. Conclusions

Myositis-related ILD presents with a wide variety of lesions, ranging from chronic to acute. It can be divided into two main forms by the types of onset, namely, chronic to subacute type showing NSIP or NSIP with an OP/FOP pattern and acute type showing ALI to DAD pattern. Anti-ARS Ab-positive cases mainly show an NSIP or FOP pattern, whereas Anti-MDA5 Ab-positive cases show ALI to DAD pattern. The early detection of findings that indicate a rapid progressive course is vital. Diffuse cranio-caudal distribution and multiple GGOs with random distribution highly likely indicate a poorer prognosis.

## Figures and Tables

**Table 1:** Common imaging findings based on types of onset.

Chronic to Subacute	Acute
Subacute course of disease > chronic	Acute or subacute onset, often with a rapid progressive course
Predominantly in the lower lobes of both lungs	Diffuse, or diffuse with a predominance of the lower lung zone
Peribronchovascular bundle distribution	Peribronchovascular distribution in the lower lobes, with parallel extension to the pleura
Reticular shadows and consolidation are common	GGO and consolidation
Reduced volume of the lower lobes	Basal volume loss
Traction bronchiectasis	

GGO: ground-glass opacification.

**Table 2:** Common imaging findings based on the antibodies.

Anti-ARS Antibody Positive	Anti-MDA5 Antibody Positive
Chronic to subacute onset >> Acute/rapidly progressive	Acute or subacute onset, often rapidly progressive
Distribution along the bronchovascular bundles in the bilateral lower lung fields > diffuse	Diffuse, predominantly in the lower lung fields, or both
Reduced volume of the lower lobes	Peribronchovascular lesion of the periphery of lower lobes, with parallel spread to the pleura
Reticular shadows or consolidation > GGO	Patchy distribution
Good response to treatment but relapses	GGO and consolidation

GGO: ground-glass opacification.

## Data Availability

Not applicable.
